# Plasma proteome profiling identifies changes associated to AD but not to FTD

**DOI:** 10.1186/s40478-022-01458-w

**Published:** 2022-10-22

**Authors:** R. Babapour Mofrad, M. del Campo, C. F. W. Peeters, L. H. H. Meeter, H. Seelaar, M. Koel-Simmelink, I. H. G. B. Ramakers, H. A. M. Middelkoop, P. P. De Deyn, J. A. H. R. Claassen, J. C. van Swieten, C. Bridel, J. J. M. Hoozemans, P. Scheltens, W. M. van der Flier, Y. A. L. Pijnenburg, Charlotte E. Teunissen

**Affiliations:** 1 Neurochemistry Laboratory and Biobank, Department of Clinical Chemistry, Amsterdam Neuroscience, VU University Medical Center, Amsterdam UMC, Vrije Universiteit Amsterdam, PO Box 7057, 1007 MB Amsterdam, The Netherlands; 2 Alzheimer Center and Department of Neurology Amsterdam, Department of Neurology, Neuroscience Campus Amsterdam Neuroscience, VU University Medical Center, Amsterdam UMC, Vrije Universiteit Amsterdam, Amsterdam, The Netherlands; 3 Departamento de Ciencias Farmacéuticas y de la Salud, Facultad de Farmacia, Universidad San Pablo-CEU, CEU Universities, Madrid, Spain; 4 Barcelonaβeta Brain Research Center (BBRC), Pasqual Maragall Foundation, Barcelona, Spain; 5 Department of Epidemiology and Biostatistics, VU University Medical Center, Amsterdam UMC, Vrije Universiteit Amsterdam, Amsterdam, The Netherlands; 6 Mathematical and Statistical Methods Group (Biometris), Wageningen University and Research Wageningen, Wageningen, The Netherlands; 7 Alzheimer Center Erasmus MC and Department of Neurology, Erasmus Medical Center, Rotterdam, The Netherlands; 8 Alzheimer Center Rotterdam and Department of Neurology, Erasmus University Medical Center Rotterdam, Rotterdam, The Netherlands; 9 Alzheimer Center Limburg, Department of Psychiatry and Neuropsychology, School for Mental Health and Neuroscience, Maastricht University, Maastricht, The Netherlands; 10 Institute of Psychology, Health, Medical and Neuropsychology Unit, Leiden University, Leiden, the Netherlands; 11 Department of Neurology, Leiden University Medical Centre, Leiden, The Netherlands; 12 Laboratory of Neurochemistry and Behavior, Department of Biomedical Sciences, Institute Born-Bunge, University of Antwerp, Antwerp, Belgium; 13 Department of Neurology and Alzheimer Center, University Medical Center Groningen, University of Groningen, Groningen, The Netherlands; 14 Department of Geriatric Medicine, Radboud University Medical Center, Radboudumc Alzheimer Center, Donders Institute for Brain, Cognition and Behaviour, Nijmegen, The Netherlands; 15 Department of Pathology, Amsterdam University Medical Centers Location VUmc, Amsterdam, The Netherlands

**Keywords:** Plasma biomarkers, Frontotemporal dementia, FTD, Alzheimer’s disease, AD, Somascan

## Abstract

**Background:**

Frontotemporal dementia (FTD) is caused by frontotemporal lobar degeneration (FTLD), characterized mainly by inclusions of Tau (FTLD-Tau) or TAR DNA binding43 (FTLD-TDP) proteins. Plasma biomarkers are strongly needed for specific diagnosis and potential treatment monitoring of FTD. We aimed to identify specific FTD plasma biomarker profiles discriminating FTD from AD and controls, and between FTD pathological subtypes. In addition, we compared plasma results with results in post-mortem frontal cortex of FTD cases to understand the underlying process.

**Methods:**

Plasma proteins (n = 1303) from pathologically and/or genetically confirmed FTD patients (n = 56; FTLD-Tau n = 16; age = 58.2 ± 6.2; 44% female, FTLD-TDP n = 40; age = 59.8 ± 7.9; 45% female), AD patients (n = 57; age = 65.5 ± 8.0; 39% female), and non-demented controls (n = 148; 61.3 ± 7.9; 41% female) were measured using an aptamer-based proteomic technology (SomaScan). In addition, exploratory analysis in post-mortem frontal brain cortex of FTD (n = 10; FTLD-Tau n = 5; age = 56.2 ± 6.9, 60% female, and FTLD-TDP n = 5; age = 64.0 ± 7.7, 60% female) and non-demented controls (n = 4; age = 61.3 ± 8.1; 75% female) were also performed. Differentially regulated plasma and tissue proteins were identified by global testing adjusting for demographic variables and multiple testing. Logistic lasso regression was used to identify plasma protein panels discriminating FTD from non-demented controls and AD, or FTLD-Tau from FTLD-TDP. Performance of the discriminatory plasma protein panels was based on predictions obtained from bootstrapping with 1000 resampled analysis.

**Results:**

Overall plasma protein expression profiles differed between FTD, AD and controls (6 proteins; *p* = 0.005), but none of the plasma proteins was specifically associated to FTD. The overall tissue protein expression profile differed between FTD and controls (7-proteins; *p* = 0.003). There was no difference in overall plasma or tissue expression profile between FTD subtypes. Regression analysis revealed a panel of 12-plasma proteins discriminating FTD from AD with high accuracy (AUC: 0.99). No plasma protein panels discriminating FTD from controls or FTD pathological subtypes were identified.

**Conclusions:**

We identified a promising plasma protein panel as a minimally-invasive tool to aid in the differential diagnosis of FTD from AD, which was primarily associated to AD pathophysiology. The lack of plasma profiles specifically associated to FTD or its pathological subtypes might be explained by FTD heterogeneity, calling for FTD studies using large and well-characterize cohorts.

**Supplementary Information:**

The online version contains supplementary material available at 10.1186/s40478-022-01458-w.

## Background

Frontotemporal Dementia (FTD) is one of the most prevalent forms of young onset dementia (< 65 years) [1]. The underlying pathological process is Frontotemporal Lobar Degeneration (FTLD), which can be mainly classified into two different pathological subtypes based on the typical protein aggregates present in brain tissue: the microtubule associated protein Tau (FTLD-Tau) or TAR DNA-binding protein 43 (FTLD-TDP) [2, 3]. Each pathological subtype will likely require distinct targeted drugs, and therefore, it is necessary to discriminate both subtypes in living patients. The poor correlation between the clinical presentation and underlying pathology [4] makes it hard to discriminate these pathological subtypes in sporadic FTD. However, in familial FTD cases (i.e. approximately 10–25% of cases [5]), the underlying genetic mutation is directly linked to these specific Tau or TDP pathologies. Genetic mutations in the microtubule-associated protein tau (*MAPT*) lead to FTLD-Tau pathology; while mutations in the progranulin (*GRN*), or chromosome 9 open reading frame 72 (*C9ORF72)* genes, lead to FTLD-TDP pathology [6].

Currently, there is no biomarker for the diagnosis and potential treatment response monitoring of FTD and its pathological subtypes. In addition, it is of particular importance to differentiate FTD from other dementia disorders, such as Alzheimer’s Disease (AD), or non-dementia disorders such as primary psychiatric disorders (PPD). Both PPD and AD can sometimes show similar clinical features as FTD, including language and executive function impairments [7, 8] or behavioral changes [3, 4]. Previous studies have shown promising cerebrospinal fluid (CSF) or blood biomarker alterations in FTD compared to controls, in particular neurofilament light (NfL) levels or the CSF p/tTau ratio for the discrimination of FTD pathological subtypes [9–12]. However, changes in these markers were either not specific for FTD as they were also changed in other types of dementia [9, 10], or did not reach sufficiently high diagnostic accuracy [11, 12]. This warrants the identification of novel biomarker candidates for diagnosis and treatment monitoring of FTD and its pathological subtypes.

Most FTD biomarker studies performed to date have used CSF as the main source for biomarker discovery, due to its close proximity to the brain [9]. However, as a lumbar puncture is often perceived as invasive, biomarkers in a more easily accessible body fluid such as blood is essential. The high-throughput multiplex aptamer-based proteomic technology (SomaScan) [13–15], able to measure > 1000 proteins in a small volume of plasma, allows for the discovery of novel blood-based biomarkers, and has been used to identify novel candidate biomarkers for AD pathology [16–18]. The multiplex feature of the aptamer-based proteomics technology is of importance as it is expected that a specific combination of proteins rather than a single biomarker will probably provide a more accurate profile of each specific dementia type, due to the complexity and heterogeneity of dementia pathologies [10].

In this study, we aimed to identify novel plasma protein profiles for the specific discrimination of FTD from AD and controls, as well as FTLD pathological subtypes using this innovative aptamer-based proteomic approach. To understand the possible relation of the different markers with the central nervous system, the plasma proteome differences were compared to those observed in post-mortem frontal cortex of FTD cases and controls.

## Methods

### Samples

#### Blood plasma

Human plasma samples from FTD subjects (*n* = 56) were obtained from two specialized memory centers in the Netherlands: Alzheimer Center Amsterdam (*n* = 96), and Erasmus Medical Center Rotterdam (*n* = 51) [19–21]. All 56 FTD subjects had a definite diagnosis of FTD based on known FTD-causing mutations (i.e. *GRN, MAPT* or *C9orf72*) and/or autopsy-confirmation. Underlying FTLD-TDP pathology was present in 40 subjects (18 autopsy confirmed cases, 13 *GRN* [of whom 1 was autopsy confirmed], 9 *C9orf72* [of whom 1 was autopsy confirmed]), and FTLD-Tau pathology in 16 subjects (3 autopsy-confirmed cases, 13 *MAPT* [of whom 2 were also autopsy confirmed]). AD plasma samples (n = 57) were selected from the Parelsnoer Initiative biobank, the neurodegeneration Parel, which collected samples from the eight Academic medical centers in the Netherlands, including Alzheimer Center Amsterdam and Erasmus Medical Center Rotterdam. AD subjects were selected based on clinical diagnosis using NINCDS-ADRDA criteria [22, 23], with either CSF biomarker results concordant with AD or MTA score ≥ 2 in subjects aged < 75, and MTA score ≥ 3 in subjects aged > 75. Control plasma samples were (n = 148) were obtained from Alzheimer Center Amsterdam (n = 69), Erasmus Medical Center Rotterdam (n = 22), and the Parelsnoer Initiative biobank (n = 57). Controls were individuals with subjective cognitive decline (SCD), in whom objective cognitive and laboratory investigations were normal (i.e., criteria for MCI, dementia, or any other neurological or psychiatric disorder not fulfilled [22]. They were selected based on the performance of cognitive tests (mini mental score examination; MMSE > 26), normal CSF biomarkers (available for all the Amsterdam and Rotterdam samples, and for 26% of the Parelsnoer samples) and if no CSF biomarker data were available, stable disease course over 1 year of follow-up. AD and control cases were not autopsy confirmed. Demographic information. Distribution of the samples per center is presented in Additional file 1: Table S1.

Of note, patients and samples within the Parelsnoer initiative followed standardized clinical and biobanking protocols at time of diagnostic work-up [22], thereby minimizing potential center and biobanking effects. All samples were collected through venipuncture using Ethylenediaminetetraacetic acid (EDTA) collection tubes. Blood collection was followed by centrifugation at 1800*g*. Plasma supernatant was collected, aliquoted and stored in 0.5 ml polypropylene tubes at − 80 °C within 4 h in each local biobank. Latest guidelines for blood sample handling for amyloid biomarker analysis recommend processing of centrifuging samples within 3 h if samples are kept at room temperature, which is in line with the targeted conditions in this study [24].

#### Post-mortem brain tissue

Post-mortem brain material was obtained from the Netherlands Brain Bank (Amsterdam, the Netherlands). We selected snap frozen medial frontal gyrus from FTD cases (FTLD-Tau n = 5; FTLD-TDP n = 5) and non-demented controls (n = 4). Four FTLD-TDP cases were familial (*GRN* n = 2, *C9orf72* n = 2) and one was a sporadic case. Of the FTLD-Tau cases, all were familial and had an underlying *MAPT* mutation. Neuropathological evaluation and processing were performed as previously described [25]. The distribution and the density of tau aggregates and TDP-43 inclusions were evaluated according to the criteria described by Lee, Cairns and MacKenzie [26–28]. Post-mortem frontal cortex was homogenized using Tissue Protein Extraction Reagent (T-Per, 0.1 g/ml, Thermo Scientific, Waltham, USA) containing EDTA-free Protease Inhibitor Cocktail (1:25, Roche, Basel, Germany), and left for 15 min at 4 °C. Homogenates were subsequently centrifuged at 10,000*g* for 15 min at 4 °C. Protein concentration was measured using Bio-Rad Protein Assay (Bio-Rad, Hercules, USA) and bovine serum albumin (BSA) (Thermo Scientific, Waltham, USA) following manufacturer’s recommendations. Samples were stored at − 80 °C until further analysis.

### Protein measures

Protein concentrations of 1303 human proteins in plasma of AD and FTD patients or controls, and brain tissue homogenates of FTD patients and controls were measured at the Neurochemistry Laboratory of Amsterdam UMC using SomaScan (SomaLogic, Inc. Boulder, Colorado, USA). Samples were diluted into three concentrations (i.e. 40%, 1%, and 0.005%) to enable the appropriate measurement range for all Somamers within one sample. The least concentrated sample is designed to detect the most abundant proteins, and the most concentrated sample is designed to detect the least abundant proteins. The precise SomaScan principle has been described in detail previously [13, 16]. Samples were randomly divided over the plates to ensure an even mix of diagnostic groups. Plasma samples were measured in 5 (AD vs. CN) and 7 (FTD vs. CN) runs, and tissue samples in 1 (FTD vs. CN) run. Technicians trained and certified by SomaLogic conducted all analyses in a blinded manner. Both plasma datasets (i.e. FTD vs. CN and AD vs. CN) were run in two batches using different SOMAmer reagent master mixes and were standardized to a common reference using common calibrator control lots. In addition, all SomaScan data were normalized following a standard three step procedure [(1) hybridization normalization, (2) plate scaling, (3) median signal normalization] to remove systematic biases in the raw assay data. No specific center effects on the overall protein expression profile were detected after principal component analysis (Additional file 2: Fig. S1).

### Statistical analysis

All statistical analyses were performed using R version 3.5.2. Demographics were compared between groups using analysis of variance (ANOVA) and Kruskal–Wallis tests where appropriate. First, we used the global test [29], which tests if the overall protein abundance profile is notably different between diagnoses. This test is suitable when there may be insufficient power to detect individual proteomic markers. We applied global testing corrected for age and sex to identify an overall difference in plasma and post-mortem protein expression profile between (1) FTD, AD and controls, and (2) FTD pathological subtypes (FTLD-Tau vs. FTLD-TDP). We also applied the global test in tissue to measure overall differences in protein expression profiles between FTD and controls. Multiplicity correction using the false discovery rate (FDR) was applied within each global test to the significant subtree that identifies those of the initial 1303 features to which the test result is attributable. FDR values < 0.05 were considered significant. Next, logistic lasso regression (LLR) with correction for age and sex was performed to select a panel of proteins that could discriminate between FTD versus controls, FTD versus AD and FTLD-Tau versus FTLD-TDP. Predictive performance was assessed by receiver operating characteristic (ROC) curves and the area under the ROC curves (AUCs). ROC curves and AUCs were produced by bootstrapping with 1000 resampling. 95% confidence interval around the resulting AUCs was calculated based on the resampling quantiles (percentile method).

## Results

### Demographics

FTD patients and controls included in the plasma analyses were both younger than AD patients, and both dementia groups had lower MMSE scores than controls (*p* < 0.05, Table 1). FTLD-Tau and FTLD-TDP subtypes did not differ in age, sex or MMSE scores. In patients selected for the tissue analysis, no differences were observed in age and sex.

**Table 1:** Demographics

	n (%)	Age, years mean (SD)^a^	Sex, female, n (%)	MMSE, mean (SD)	Post-mortem delay mean (SD) in hours
*Plasma*					
FTD	56 (22%)	59.4 (7.4)	25 (45%)	24 (5.2)	
FTLD-TDP^d^	40 (71%)	59.8 (7.9)	18 (45%)	24 (5.7)	
FTLD-Tau^e^	16 (29%)	58.2 (6.2)	7 (44%)	25 (3.6)	
AD	57 (22%)	65.5 (8.0)^c^	22 (39%)	23 (2.3)	
Controls	148 (57%)	61.3 (7.9)	60 (41%)	29 (1.4) ^b^	
*Post-mortem frontal cortex*					
FTD	10 (67%)	60.1 (8.0)	6 (60%)	NA	6.5 (3.7)
FTLD-TDP	5 (50%)	64.0 (7.7)	3 (60%)	NA	6.8 (3.1)
FTLD-Tau	5 (50%)	56.2 (6.9)	3 (60%)	NA	5.3 (0.6)
Controls	4 (33%)	61.3 (8.1)	3 (75%)	NA	8.8 (2.5)

Analysis of variance (ANOVA) or Kruskal–Wallis test were used as appropriate. *p* < 0.05 was considered significant

*AD* Alzheimer’s disease, *TDP* TAR DNA binding protein 43, *FTD* frontotemporal lobar degeneration, *MMSE* mini mental state examination, *NA* not available

^a^Age at inclusion in plasma samples and age at death in post-mortem tissue

^b^FTD patients and AD had significantly lower MMSE scores

^c^FTD patients and controls were significantly younger than AD patients

^d^FTLD-TDP pathology was present in 40 subjects (18 autopsy confirmed cases, 13 *GRN* [of whom 1 was autopsy confirmed], 9 *C9orf72* [of whom 1 was autopsy confirmed]), and FTLD-Tau pathology in 16 subjects (3 autopsy-confirmed cases, 13 *MAPT* [of whom 2 were also autopsy confirmed])

^e^FTLD-TDP was present in 5 subjects (4 cases were familial [*GRN* n = 2 and *C9orf72* n = 2] and 1 was a sporadic case), and FTLD-Tau was present in 5 cases (all 5 cases were familial [*MAPT* n = 5])

### Plasma protein profile differs between FTD, AD and controls.

The overall plasma protein expression profile consisting of 1303 proteins was different between FTD, AD patients and controls (*p* = 0.005). We identified six proteins that attributed to this difference in expression profile (FN1.3, Fibronectin, FN1.4, VWF, ECM1 and ApoE; Table 2, Additional file 2: Fig. S2), which were all upregulated in AD compared to both FTD patients and controls (Table 2). Proteins specifically associated to FTD were not detected. There was no difference in overall plasma protein profiles between FTD-Tau and FTLD-TDP subtypes (*p* > 0.05).

**Table 2:** Plasma proteins from global test comparing FTD, AD and controls

Name	Associated with status	Statistic^a^	Std. dev	*p* value
FN1.3	AD	8.72	0.406	2.26e−10
Fibronectin	AD	7.39	0.405	5.07e−09
FN1.4	AD	7.18	0.406	2.04e−08
VWF	AD	7.01	0.407	3.65e−08
ECM1	AD	4.23	0.398	1.94e−05
ApoE	AD	5.68	0.406	9.79e−07

Plasma proteins that attributed to a difference in expression profile between FTD, AD and controls after correction for multiple testing

All proteins were upregulated in AD compared to FTD and controls. Models were corrected for age and sex

*FN 1.3* fibronectin fragment 3, *FN 1.4* fibronectin fragment 4, *VWF* Von Willebrand factor, *ECM1* extracellular matrix protein 1, *ApoE* apolipoprotein epsilon, *AD* Alzheimer’s disease

^a^Represent the t statistics associated to the Global testing

### Plasma protein profiles can discriminate FTD from AD, but not between pathological subtypes

**Fig. 1:**
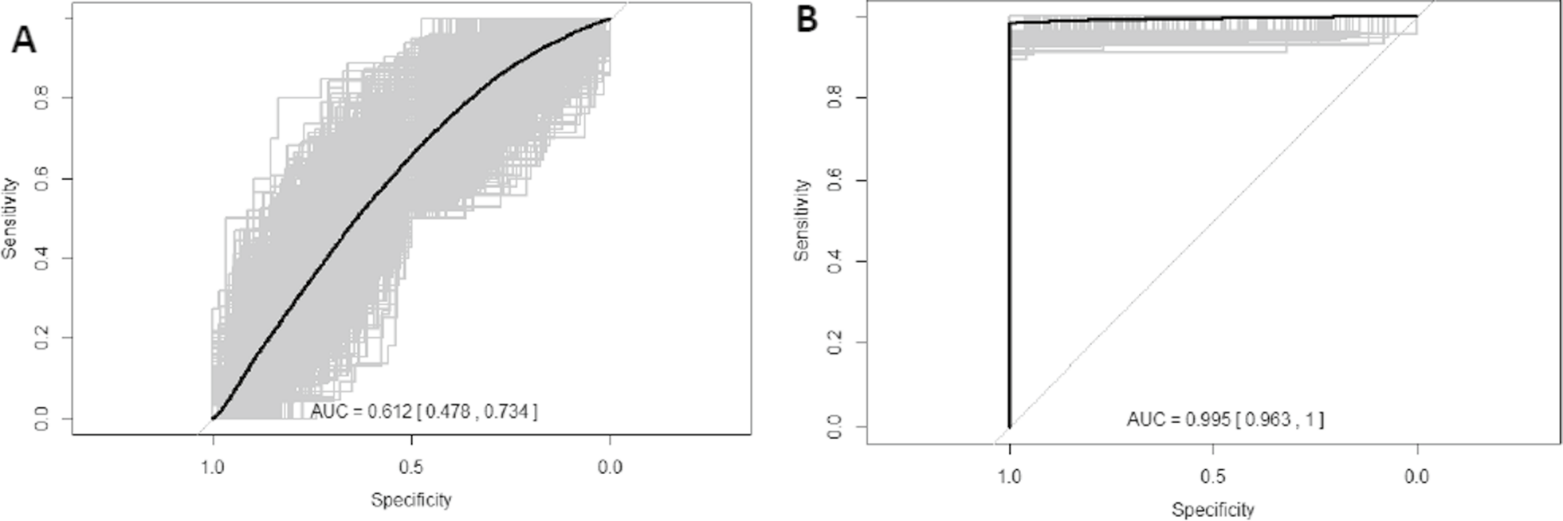
Receiver operating characteristic (ROC) curves discriminating FTD from controls or AD based on plasma protein sets. Black line is the mean area under the curve (AUC) after bootstrapping with 1000 resampling. **A** FTD versus controls, **B** FTD versus AD. *FTD* frontotemporal dementia, *AD* Alzheimer’s disease

Next, we set out to identify panels of plasma proteins to discriminate between FTD versus Controls, FTD versus AD and FTD-Tau versus FTLD-TDP. No plasma proteomic signal that reliable discriminated FTD from controls or FTD pathological subtypes was detected (Fig. 1, AUC:0.61; 95% CI 0.48–0.73). We however identified a panel of 12 plasma proteins that discriminated FTD from AD with very high accuracy (AUC: 0.99, 95% CI 0.96–1) (Fig. 1; Table 3).

**Table 3:** Classification protein panel for FTD versus AD

Protein name	Beta^a^
FN1.3	− 3.390
Fibrinogen gamma chain dimer	− 1.315
hnRNPK	− 0.575
vWF	− 0.500
IDS	− 0.276
RSPO3	− 0.168
STRATIFIN (14-3-3 protein sigma)	− 0.098
TS	− 0.077
IL24	− 0.074
TRY3	− 0.014
FN1.4	− 0.006
TPSG1	0.003

Plasma proteins for the discrimination of FTD versus AD. Model did not select for age and sex. Inclusion. Of age and sex did not modify the outcomes

*FTD* frontotemporal dementia, *AD* Alzheimer’s disease, *RSPO3* R-spondin 3, *VWF* Von Willebrand factor, *IDS* iduronate 2-sulfatase, *IL24* interleukin 24, *TPSG1* tryptase gamma 1, *FN 1.3* fibronectin fragment 3, *FN 1.4* fibronectin fragment 4, *TRY3* serine protease 2, *HNRNPK* heterogeneous nuclear ribonucleoprotein K, *TS* thymidylate synthetase

^a^Reflect the effect size of the corresponding protein to the classification signature. Reference is FTD

### Tissue proteins levels differ between FTD subjects and controls, but not between pathological subtypes

Next, we exploratory analyzed post-mortem brain tissue of FTD cases versus controls, and FTD subtypes. The overall tissue protein expression profile was different between FTD and controls (*p* = 0.003). We identified seven proteins that attributed to this difference in expression profile, of which four were upregulated (C4, Discoidin domain receptor 1, Annexin I, Alpha-1-antichymotrypsin complex, Table 4) and three were downregulated in FTD (WIF 1, LRRT3, HO 2, Table 4). Similar to plasma results, no differences in overall brain protein profile was detected between FTD-Tau and FTD-TDP subtypes (*p* > 0.05).

**Table 4:** Tissue proteins from global test

Name	Associated with status	Statistic^a^	Std. dev	*p* value
C4	FTD	85.4	10.5	2.30e−06
WIF 1	Control cortex	80.6	10.5	1.30e−05
Discoidin domain receptor 1	FTD	80.4	10.5	1.42e−05
LRRT3	Control cortex	78.0	10.5	2.84e−05
HO 2	Control cortex	71.9	10.5	1.29e−04
Annexin I	FTD	61.7	10.5	8.67e−04
Alpha-1-antichymotrypsin complex	FTD	65.7	10.5	4.39e−04

Tissue proteins that attributed to a difference in expression profile between FTD and control tissue after correction for multiple testing. Proteins were associated with either FTLD or control cortex

*C4* Complement C4, *WIF-1* Wnt inhibitory factor 1, *LRRT3* Leucine-rich repeat transmembrane neuronal protein 3, *HO-2* Heme oxygenase 2, *FTD* Frontotemporal dementia

^a^Represent the t statistics associated to the Global testing

## Discussion

In this plasma proteomics study, we measured 1303 proteins in over 260 human plasma samples to identify protein profiles for the specific diagnosis of FTD and its pathological subtypes. We found a difference in overall protein profile between FTD, AD and controls, but none of the plasma proteins was specifically associated to FTD. Importantly, we identified a plasma protein panel that discriminated FTD from AD patients, but not FTD from controls. No plasma or tissue protein changes were detected between FTD pathological subtypes.

To our knowledge, we were the first to apply proteomics in blood plasma of genetically or pathologically confirmed FTLD patients [30]. We found a difference in overall plasma protein profiles between FTD, AD patients and controls, which could be attributed to six proteins. All these proteins were upregulated in AD compared to FTD patients and controls, which suggests that none of the proteins identified are associated to FTD pathogenesis. In line with these findings, our bootstrap classification exercises identified a combination of 61 proteins demarcating FTD patients and non-demented controls with limited accuracy (AUC: 0.61) and large confidence intervals, underpinning the insufficient diagnostic accuracy and further supporting the lack of specific plasma protein signals specifically associated to FTD. These results contrast with previous unbiased proteomics studies performed in CSF samples, including ours, in which several CSF FTD biomarker candidates were identified [31, 32]. Whether such discrepancies are driven by the different technologies (i.e. aptamer based protein array vs. unbiased mass spectrometry) or the different biology underlying these matrices (CSF vs. plasma) remains to be established. Current high-throughput proteome arrays (e.g. aptamer based, proximity ligation assays) are dependent on the protein library used, and thus may fail to capture the full proteome differences detected by unbiased mass spectrometry studies. Still, several proteomics studies have already highlighted the low overlap and correlations between CSF and plasma proteomes even when the same technology is used [33, 34]. Both factors, i.e. technological and biological matrix bias, likely contribute to the discrepancies with previous CSF FTD proteomics studies. Considering that the number of FTD and AD cases analyzed were comparable, the lack of plasma biomarker signals associated to FTD might be also partly explained by the clinicopathological diversity of FTD. The different clinical, genetic and pathological phenotypes within the FTD spectrum may hurdle the identification of specific biomarkers, highlighting the need to include large cohorts in biomarker studies [35].

We identified a panel of 12 blood-based proteins discriminating FTD from AD with very high accuracy (AUC: 0.99). Three of these proteins [fibronectin fragments 3 and 4 and Von Willebrand Factor (vWF)], were among the proteins differentially regulated between AD, FTD and controls identified before. Our findings are supported by a previous AD aptamer-based study, where fibronectin fragment 4 and fibronectin were also selected in a panel of plasma proteins to discriminate AD patients from controls [16]. The observed high diagnostic accuracy supports potential use of this blood-based biomarker panel for the differential dementia diagnosis. However, as an AUC of 0.99 is near to perfect, replication of these findings, preferably through external validation is needed. The four proteins that showed the strongest effect on demarcating FTD from AD (largest beta coefficients) and thereby contributed most to the discriminatory panel, namely Fibronectin, Fibrinogen gamma chain, hnRNPK and vWF, will be discussed in more detail. The protein with the strongest beta was Fibronectin (FN), a glycoprotein that plays a role in tissue repair, and regulating cell attachment, motility, hemostasis and embryogenesis [36]. Several studies reported higher amounts of high molecular FN forms in plasma, CSF and frontal and temporal cortex of AD patients compared to vascular dementia and controls [37–39], corroborating our results showing higher levels of fibronectin fragments 3 and 4 in AD patients compared to FTD patients and controls. Interestingly, increased expression of FN type III domain has shown to decrease Aβ secretion in a cellular model [40]. These data together suggest an increase of fibronectin fragments in AD which might potentially convey a neuroprotective effect. The protein with the second highest beta was Fibrinogen gamma chain, a blood borne glycoprotein essential to form an insoluble fibrin matrix. It is associated to amyloid deposition [41] and brain atrophy [42]. The lower levels of this protein in AD compared to FTD and controls [43] indicate that this marker is specifically associated to AD pathogenesis. Experimental and neuropathological studies indeed suggest that this protein may contribute to AD by altering thrombosis and fibrinolysis [44]. hnRNP K is one of the major pre-mRNA-binding proteins, likely playing a role in the nuclear metabolism of hnRNAs and in the p53/TP53 response to DNA damage [45]. A previous proteome study found an upregulation of this protein in frontal cortex of AD cases [46]. Recent exciting evidence showed mislocalisation of hnRNA K in pyramidal neurons of the frontal cortex to be a novel neuropathological feature associated with both frontotemporal lobar degeneration and ageing [47, 48]. Future studies should therefore address the potential role of this protein in both FTD and AD to understand how it contributes to discriminate these disorders. The protein with the fourth highest beta was VWF, a glycoprotein with critical functions in hemostasis [49]. It was identified by the global test and was also part of the protein panel discriminating AD and FTD. VWF has frequently been studied in AD since vascular damage plays a role in the pathogenesis of AD dementia. However, results of VWF levels in AD patients have been conflicting. One CSF proteomics study that aimed to discriminate AD from non-AD patients has shown discrepant results in CSF VWF levels between three independent cohorts [37]. Other studies reported no difference in VWF levels in blood plasma, CSF or brain cells between AD and controls [50, 51], and one large population study reported higher levels of VWF in blood plasma of AD patients [52]. We recently observed increased levels of CSF VWF in our ongoing AD studies (Del Campo et al. under review, Additional file 2: Fig. S3). A possible speculative explanation for these discrepant findings could be that the cohorts that reported an increase in VWF levels, including ours, had more patients with mixed vascular and AD pathology, whereas other cohorts mostly included patients with pure AD pathology. It would be very relevant to investigate the markers identified here together with novel promising plasma biomarkers, such as plasma pTau levels, which shows very good discrimination between AD and FTD patients, being specifically increased in AD [53–55].

We could not find differentially regulated proteins between Tau and TDP pathological subtypes in tissue or plasma, nor could we identify discriminatory plasma protein signatures between these subtypes. Throughout literature, it has been challenging to identify and validate protein alterations between both pathological subtypes. For CSF, previous proteomic studies reported several differentially regulated proteins [31] or a biomarker panel enabling sensitive differentiation between TDP and Tau pathology [56], although independent multicenter validation and replication on different platforms is still needed. The lack of a biomarker (panel) for FTD subtypes with feasibility in clinical practice thus far, could have several possible explanations. First, a potential explanation is the heterogeneity within Tau and TDP pathological subtypes, such as the different isoforms of TDP and Tau pathology, which have not been accounted for in fluid biomarker studies so far [57, 58]. For instance, patients with the TDP-A isoform might have a different protein signature than patients with the TDP-C isoform. This heterogeneity will complicate the search for a single discriminatory protein panel for TDP versus Tau, and will require larger and more homogeneous sample sizes, which are scarce. An alternative explanation could be that both pathological subtypes might have similar downstream pathological pathways leading to FTD. For instance, local TDP and Tau pathology could potentially be initiating the same prominent cascades, represented in similar proteomic changes in body fluids, ultimately leading to the neurodegenerative changes seen in FTD. This could also explain why both pathological subtypes are seen across the clinical FTD spectrum [10]. Lastly, in most FTD biomarker studies familial and sporadic cases are often grouped to achieve a large sample size. However, the question remains whether the familial form of FTD with *GRN*, *C9orf72* or *MAPT* mutations is biologically similar to sporadic FTD patients with TDP or Tau proteins. Future studies where (plasma) protein profiles of familial and sporadic FTD subtypes are independently studied could provide more clarity.

Proteomics in body fluids such as blood plasma or CSF can provide valuable mechanistic information as to whether post-mortem pathological changes are also seen in earlier ante-mortem disease stages, or whether there are also systemic responses involved in CNS diseases. As suggested also by the Consensus report of The Reagan Working group in 1998 [59], comparison of biofluid results with the expression of those proteins in brain tissue would be the most direct proof for a relation with the brain pathology. Indeed, pathological correlates are the basis for the now widely used biomarkers in AD, such as amyloid beta and pTau. From the six proteins identified in our exploratory tissue proteome investigation, four were detected also in a recent mass spectrometry study (C4, Annexin I, Ho2 and Alpha-1-antichymotrypsin complex) [60]. In such study C4 and Annexin I were also increased or tended to be increased in FTD compared to controls. However, we observed that the proteins differentially regulated in FTD brain tissue were not dysregulated in plasma, suggesting that the changes identified in brain are not reflected in plasma. Despite the exploratory nature of these findings, the results are in line with the limited association between CSF and plasma proteomes discussed above, and more recently, between brain and plasma or serum proteomes [61, 62]. This might be explained by the redundancy of plasma proteins from the periphery, which may mask low concentration and subtle changes of CNS-derived proteins in plasma. In addition, the lack of plasma FTD changes may also suggest no systemic changes underlying FTD pathophysiology. It is important to note that biofluid based biomarker levels are dynamic and may change along the disease process [63]. Thus, the different time point of collection (i.e. ante-mortem for plasma vs. post-mortem for tissue), may also explain the lack of overlap. However, the small sample size of the tissue sections prohibits strong conclusions.

Among the limitations of our study is that despite the large number of plasma proteins analyzed, the aptamer-based proteomic platform is still a targeted analysis dependent on the protein library. Thus, we cannot exclude that other relevant or powerful brain-disease related biomarkers are not present within the aptamer library (i.e. Somamer library [64]). Nevertheless, the hypothesis free approach allowed us to identify novel proteins in addition to previously described proteins. Another limitation is that there was some center bias, because especially AD and control samples were collected from several sites (five). However, the majority (two third) of the AD samples were from the two sites that provided also the FTD samples, and all centers collected their samples under the same standardized protocol. Another limitation is the lack of replication of our findings in an independent validation cohort, especially considering the high accuracy of our FTD vs AD discriminatory panel. Validation of the plasma panel is technically not feasible yet on the Somascan technology. However, some of the markers within the panel have been validated by others and also within our current CSF studies using alternative platforms (e.g., vWF and RSPO3, Additional file 2: Fig. S3. Del Campo et al. under review). Novel large proteomics studies using an independent platform (proximity extension assay) with versatility of building smaller panels in plasma of FTD patients are current underway in the course of the JPND bPRIDE project (https://www.neurodegenerationresearch.eu/wp-content/uploads/2020/05/PROJECT-bPRIDE.pdf; neurodegenerationresearch.eu). It should be noted that different type proteomic platforms can now be used for discovery of novel plasma-based biomarkers, from unbiased based mass-spectrometry platforms to targeted high throughput proteome arrays. All have their own pros and cons in relation to protein coverage, sensitivity, specificity, dynamic range, or translatability into diagnostic assays [35, 65].

The strengths of our study are that all our FTD cases had confirmed diagnosis based on genetic and/or pathological confirmation. Because FTD is clinically heterogeneous and does not correlate strongly to its pathologic subtypes, cohorts with known pathologic subtypes are important to provide relevant insights into underlying disease mechanisms. Of note, some of the AD plasma samples analyzed in this study came from non-specialized memory clinics and were diagnosed using clinical criteria without AD CSF biomarker confirmation.

## Conclusions

In summary, we analyzed an unprecedented large number of proteins (1303) in plasma of FTD cases with confirmed underlying neuropathology together with AD and cognitively unimpaired controls. We observed that the plasma or tissue proteome were essentially similar between FTLD-Tau and FTLD-TDP. When the overall FTD group was analysed, we identified six plasma proteins differentially regulated between AD, FTD, and controls. However, these were primarily associated to AD dementia rather than FTD, underpinning the challenges to identify robust single markers associated to FTD. Classification exercises revealed a plasma protein panel discriminating FTD from AD with high accuracy, which needs to be validated in independent cohorts. Once validated, FTD should only be considered when the protein biomarker results are compatible with the clinical presentation, as these proteins might be useful to exclude AD, rather than to specifically detect FTD pathophysiology. Further studies should show if these markers are useful to differentiate FTD from psychiatric disorders, such as observed for NfL [11, 66–68]. The lack of plasma protein signals specifically associated to FTD-confirmed cases might be caused by the heterogeneity of this disorder, highlighting that the quest of FTD-specific biomarkers will likely require high-collaborative biomarker studies using large and well-characterized FTD cohorts [35]. Such heterogeneity will likely hamper the identification of a single FTD-specific biofluid marker. Thus, measurements of additional matrices or targets (e.g., RNA, extracellular vesicles), integration of multi-omics approaches, system-based analysis and/or the development of computer assisted algorithms will likely be needed to capture the full complexity FTD and its pathological subtypes [35, 60, 69, 70].

## Supplementary Information

**Additional file 1. Table S1.** Distribution of diagnoses over the different centers.

**Additional file 2**.

## Data Availability

The datasets generated and/or analysed during the current study are not publicly available yet, but will be made available once the paper is accepted.
