# Supplementary material for: Plasma proteome profiling identifies changes associated to AD but not to FTD
Source: Acta Neuropathol Commun. 2022 Oct 22;10:148. doi: 10.1186/s40478-022-01458-w (PMC9587555; doi:10.1186/s40478-022-01458-w)
Supplement: Supplementary file 1 — Additional file 1. Table S1. Distribution of diagnoses over the different centers. [file 40478_2022_1458_MOESM1_ESM.docx]

| Additional file 1: Table S1. Distribution of diagnoses over the different centers | | | | |  |  |
| --- | --- | --- | --- | --- | --- | --- |
| Diagnostic group | Center 1 | Center 2 | Center 3 | Center 4 | Center 5 | Center 6 |
| SCD-AD | 14 | 10 | 5 | 10 | 4 | 14 |
| SCD-FTD | 69 | 22 | 0 | 0 | 0 | 0 |
| AD | 19 | 18 | 1 | 7 | 1 | 10 |
| FTD | 27 | 29 | 0 | 0 | 0 | 0 |
|  | | | | |  |  |

Number of samples distributed per center. SCD, Subjective cognitive decline (SCD-FTD or SCD-AD depending on whether they were measured in the same run as the FTD or AD samples respectively; AD, Alzheimer´s disease; FTD; Frontotemporal Dementia)
