# Supplementary material for: Plasma proteome profiling identifies changes associated to AD but not to FTD
Source: Acta Neuropathol Commun. 2022 Oct 22;10:148. doi: 10.1186/s40478-022-01458-w (PMC9587555; doi:10.1186/s40478-022-01458-w)
Supplement: Supplementary file 2 — Additional file 2. [file 40478_2022_1458_MOESM2_ESM.pptx]

## Slide 1
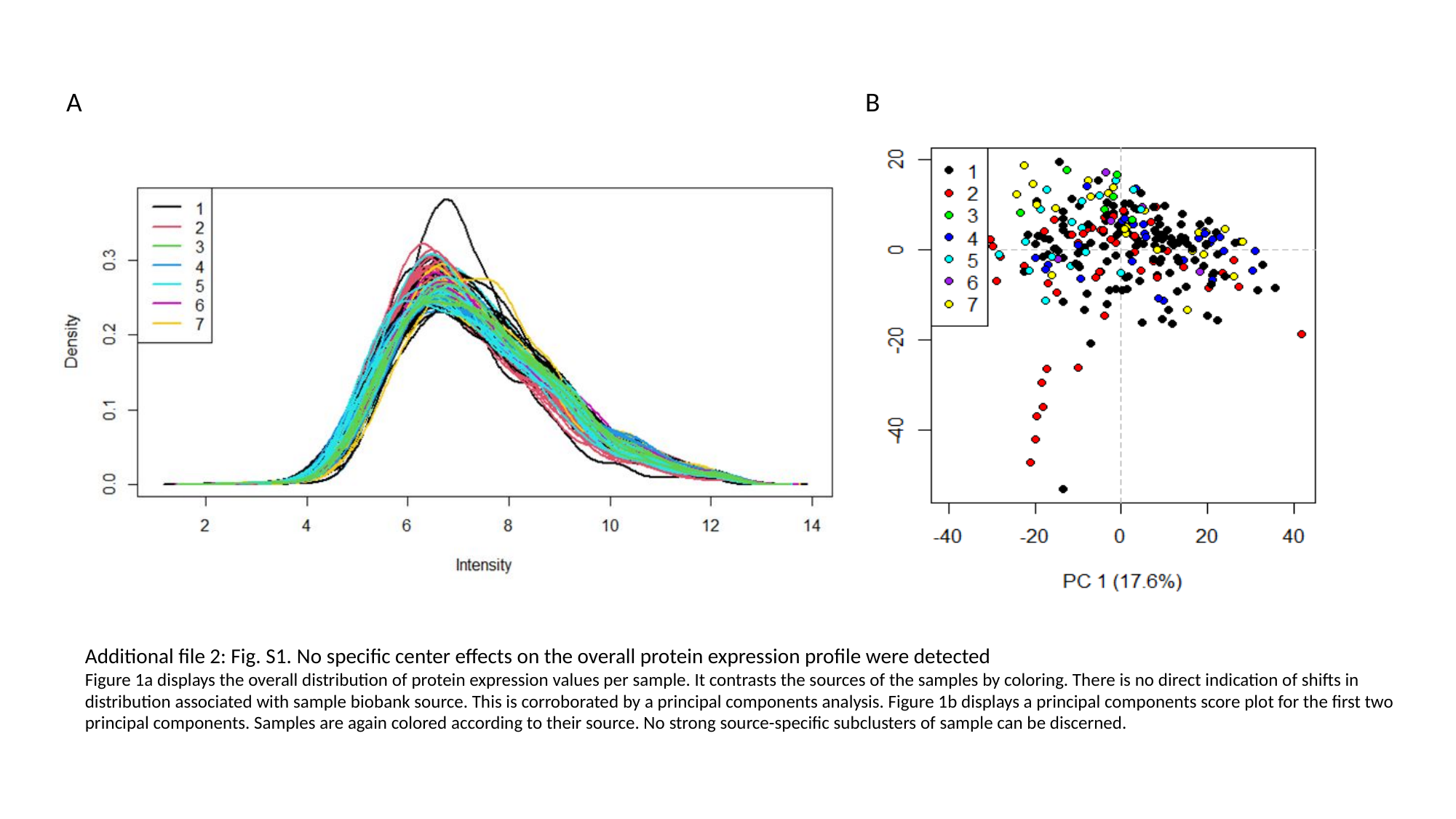

A
B
Additional file 2: Fig. S1. No specific center effects on the overall protein expression profile were detected
Figure 1a displays the overall distribution of protein expression values per sample. It contrasts the sources of the samples by coloring. There is no direct indication of shifts in distribution associated with sample biobank source. This is corroborated by a principal components analysis. Figure 1b displays a principal components score plot for the first two principal components. Samples are again colored according to their source. No strong source-specific subclusters of sample can be discerned.

## Slide 2
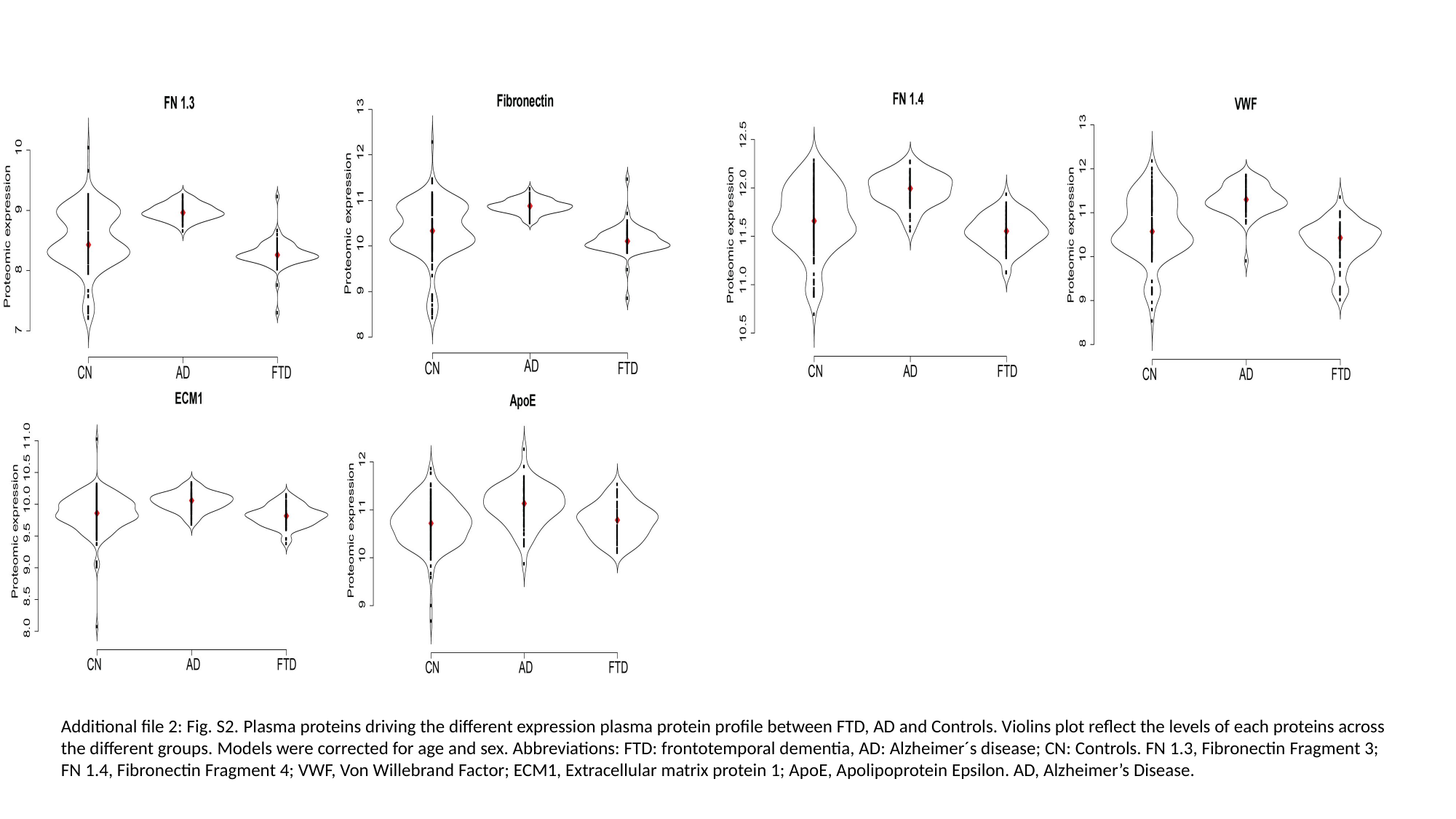

Additional file 2: Fig. S2. Plasma proteins driving the different expression plasma protein profile between FTD, AD and Controls. Violins plot reflect the levels of each proteins across the different groups. Models were corrected for age and sex. Abbreviations: FTD: frontotemporal dementia, AD: Alzheimer´s disease; CN: Controls. FN 1.3, Fibronectin Fragment 3; FN 1.4, Fibronectin Fragment 4; VWF, Von Willebrand Factor; ECM1, Extracellular matrix protein 1; ApoE, Apolipoprotein Epsilon. AD, Alzheimer’s Disease.

## Slide 3
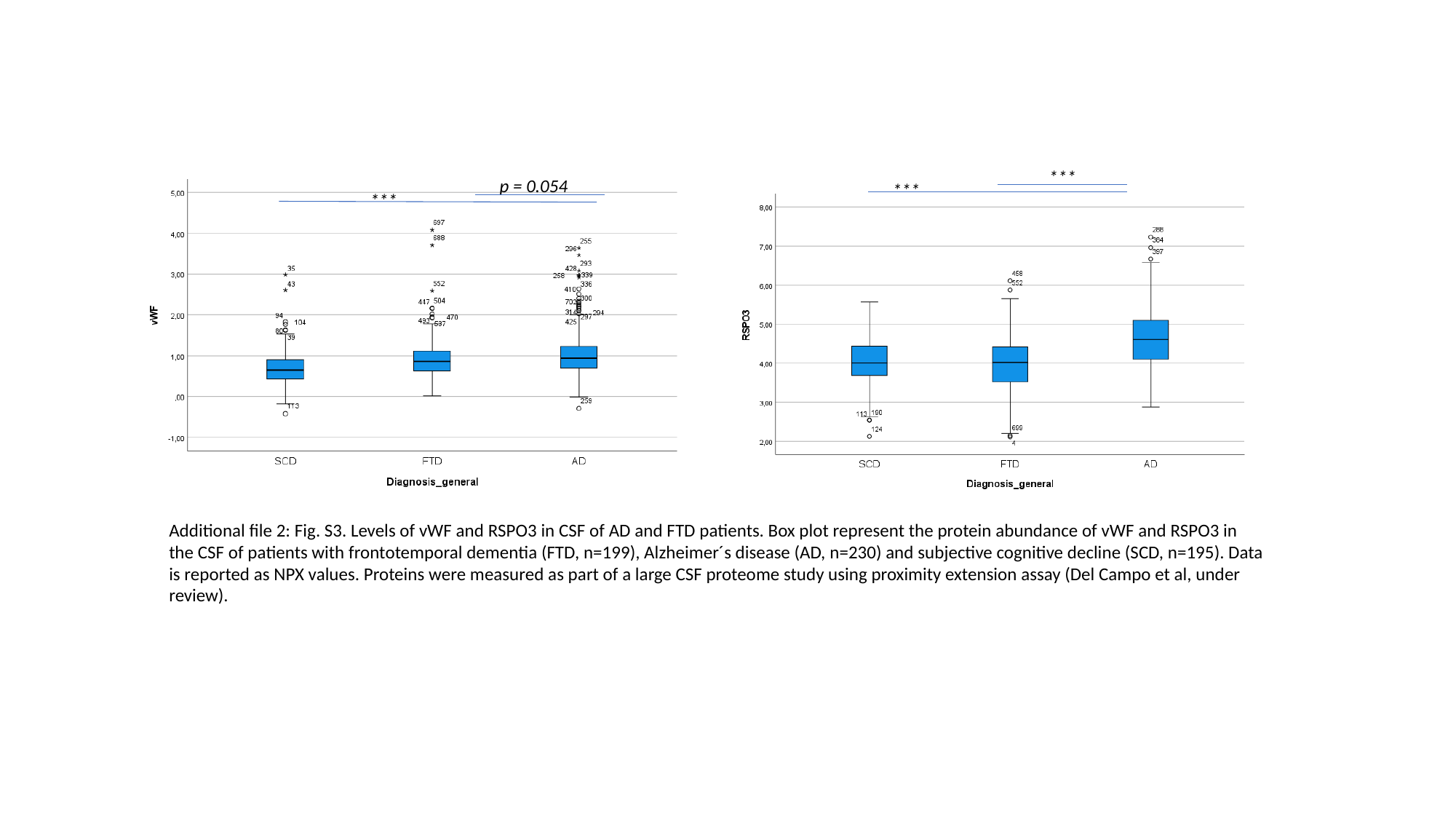

***
 p = 0.054
***
***
Additional file 2: Fig. S3. Levels of vWF and RSPO3 in CSF of AD and FTD patients. Box plot represent the protein abundance of vWF and RSPO3 in the CSF of patients with frontotemporal dementia (FTD, n=199), Alzheimer´s disease (AD, n=230) and subjective cognitive decline (SCD, n=195). Data is reported as NPX values. Proteins were measured as part of a large CSF proteome study using proximity extension assay (Del Campo et al, under review).
